# The Impact of Gynecological Pathologies on Patients’ Quality of Life from Menarche to Menopause—Literature Review

**DOI:** 10.3390/jcm14176281

**Published:** 2025-09-05

**Authors:** Mihaela Amza, Bashar Haj Hamoud, Romina-Marina Sima, Gabriel-Petre Gorecki, Mircea-Octavian Poenaru, Andrei-Sebastian Diaconescu, Nicolae Gică, Ancuta-Alina Constantin, Mihai Popescu, Liana Pleș

**Affiliations:** 1 Department Ph.D., IOSUD, “Carol Davila” University of Medicine and Pharmacy, 020021 Bucharest, Romania; mihaela.amza@umfcd.ro; 2 Department of Obstetrics and Gynecology, “Carol Davila” University of Medicine and Pharmacy, 020021 Bucharest, Romania; mircea.poenaru@umfcd.ro (M.-O.P.); gica.nicolae@umfcd.ro (N.G.); liana.ples@umfcd.ro (L.P.); 3 “Bucur” Maternity, Saint John Hospital, 012361 Bucharest, Romania; 4 Department of Obstetrics and Gynecology, Faculty of Medicine, Saarland University Hospital, 66424 Homburg, Germany; bashar.hajhamoud@uks.eu; 5 Faculty of Medicine, Titu Maiorescu University, 040441 Bucharest, Romania; gabriel.gorecki@prof.utm.ro; 6 Department of General Surgery, Faculty of Medicine, “Carol Davila” University of Medicine and Pharmacy, 050474 Bucharest, Romania; andrei.diaconescu@umfcd.ro; 7 General Surgery Department, Fundeni Clinical Institute, 022328 Bucharest, Romania; 8 Filantropia Clinical Hospital Bucharest, 011132 Bucharest, Romania; 9 Department of Cardio-Thoracic Pathology, “Carol Davila” University of Medicine and Pharmacy, 020021 Bucharest, Romania; ancuta-alina.constantin@umfcd.ro; 10 “Marius Nasta” National Institute of Pneumology, 050159 Bucharest, Romania; 11 Department of Anaesthesia and Intensive Care, “Carol Davila” University of Medicine and Pharmacy, 020021 Bucharest, Romania; mihai.popescu@umfcd.ro; 12 Department of Anaesthesia and Intensive Care, Bucharest University Emergency Hospital, 050098 Bucharest, Romania

**Keywords:** quality of life, dysmenorrhea, menopause, endometriosis, infertility, uterine fibromatosis, polycystic ovary syndrome, pelvic organ prolapse

## Abstract

Quality of life represents a key area of concern in every stage of life. It is necessary to pay more attention to the factors or conditions that can negatively affect quality of life. From adolescence until postmenopause, women face a series of gynecological conditions that can significantly reduce their quality of life and which also represent economic problems. We conducted a literature review to present the impact of gynecological pathologies on patients’ quality of life and the tools used to assess these effects. The goals were to increase the attention given to this topic and to encourage health care providers to prioritize patients’ quality of life in the management of gynecological pathologies. Most of the time, gynecological pathologies reduce self-esteem, cause fear, anxiety, and depression, and the feeling of a lack of control may appear. Social life, professional and university activities, relationships, and daily or recreational activities can be negatively influenced by gynecological pathologies. Sexual life and relationships with partners are often affected by the presence of gynecological conditions, especially if they cause infertility. From the effects of dysmenorrhea that occur since the teenage and until the onset of menopause symptoms, women frequently present throughout life a series of gynecological disorders such as endometriosis, infertility, uterine fibromatosis, polycystic ovary syndrome, pelvic organ prolapse, or urinary incontinence. Understanding the negative effects that these conditions have on quality of life can contribute to more efficient and personalized management of cases.

## 1. Introduction

Quality of life (QoL) is a complex concept that is based on the individual’s perception of their position in life, taking into account the culture and environment where they live, as well as the standards and expectations they have. Quality of life depends on a series of factors such as psychological state, physical health, social relationships, level of autonomy, and professional activities [1]. Quality of life has been an intensely debated topic since the 1940s, when health professionals realized that psychological and social factors are important for health, thanks to advances in medicine that have prolonged survival [2]. Over time, several instruments have been developed to assess patients’ quality of life, such as the World Health Organization Quality of Life instrument (WHOQOL) [3,4], the Medical Outcomes Study Short Form-36 Health Survey [5], or the Duke Health Profile [6].

The World Health Organizations have emphasized the fact that attention must be paid to quality of life in every stage of life [7]. It has been reported that women have a lower quality of life than men because they are exposed to different social and cultural factors, such as different income, political, and social limitations, more family responsibilities, and difficulty accessing health services [8]. In addition, throughout life, women can face numerous gynecological conditions that have an effect on health, from menarche up to the postmenopausal period. Nowadays, nonmalignant gynecological conditions have an unclear epidemiology. Physical health and therefore quality of life can be affected by gynecological conditions such as uterine fibromatosis, adenomyosis, endometriosis, genital prolapse, or menopause [9]. Up to 90% of women face the onset of menstrual pain shortly after menarche, so their quality of life can be affected every month during the menstrual period from their teenage years [10]. Later in life, every woman experiences unpleasant symptoms of menopause, which usually occur between 45 and 55 years of age and which have negative effects on their quality of life [11].

We performed a literature review on the impact of gynecological conditions on patients’ quality of life, from the adolescent period to the postmenopausal period. Also, we present the available tools that can be used to evaluate the relationship between gynecological pathologies and quality of life. The objective of the study was to increase the attention paid to this very important and current topic and to highlight the importance of correct treatment in different gynecological conditions in order to avoid negative effects on quality of life. We present the effects of each pathology on the daily life of patients.

Nowadays, quality of life is a debated topic, and people are continuously attempting to identify the factors that negatively affect quality of life. We have reviewed the literature and selected articles that have analyzed the relationship between gynecological pathologies and quality of life in our times, which are dominated by stress and multiple activities that need to be managed. We conducted a synthesis of studies in the literature to provide a general, descriptive, and comprehensive perspective on this topic. We had a flexible approach without constraining the selection of studies by strict criteria. A possible limitation is that relevant studies in the literature may not have been included in our review.

A limitation of this literature review is that many of the questionnaires used to assess the impact of gynecological pathology on patients’ quality of life have not been translated and validated in all countries. This fact suggests that the comparability and applicability of the results across populations may be limited. Future studies are needed to adapt and validate these instruments cross-culturally. Our review provides an overview of the negative effects that gynecological pathologies can have on quality of life and presents some of the available methods of assessment.

Table 1 presents information about the most commonly used questionnaires for assessing the impact of gynecological pathology on patients’ quality of life and details about their translation and validation status in different languages.

This literature review did not specifically assess population factors such as ethnicity, culture, and religion and how they may influence symptom perception and reporting of quality of life outcomes. This is a limitation of our study and highlights the need for future studies that specifically address these variables.

## 2. Dysmenorrhea and Patients’ Quality of Life

Dysmenorrhea represents the presence of menstrual pain, one of the most common gynecological conditions in patients of reproductive age. Its worldwide prevalence varies between 16% and 91%. Severe menstrual pain was reported in 2 to 29% of cases [28,29]. Two types of dysmenorrhea were described: primary and secondary. Primary dysmenorrhea does not have an identifiable cause and appears most of the time shortly after menarche. Secondary dysmenorrhea was associated with numerous pelvic conditions such as endometriosis, uterine fibromatosis, or ovarian cysts [30,31]. The risk factors for dysmenorrhea are the familiar history of dysmenorrhea, heavy menstrual flow [32], young age at menarche, nulliparity [33], menstrual cycle irregularity, obesity, and smoking [34].

Menstrual pain usually lasts for 1–2 days and starts shortly before the onset of menstrual bleeding. In the case of young women, painful symptoms are underestimated and considered normal. Ineffective management of dysmenorrhea causes a poor quality of life for several days each month for women of reproductive age [33]. In a study that included 1349 medical students with dysmenorrhea, approximately 64.3% of them stated that dysmenorrhea affected their quality of life [35]. The same opinion was shared by 73.9% of the participants in a study that included a total of 422 women with dysmenorrhea between 18 and 45 years old. In this study by Amza et al., the DysmenQoL score was used to evaluate the impact of dysmenorrhea on quality of life. This tool proved to be a useful and valid method, but future studies are needed to reproduce its reliability and validity. The DismenoL score increased when the intensity of menstrual pain was greater [36]. Dysmenorrhea has negative effects on the quality of life of patients by affecting daily activities [37], professional or academic performance, social and couple relationships, physical or recreational activities, and sleep [38].

In an extensive study by Schoep et al., which included 42,879 women, it was observed that dysmenorrhea was present in 85% of the participants, and 1 out of 3 women included in the study stated that they used to change their daily activity schedule due to the presence of symptoms associated with menstruation [39]. Esan et al. conducted a study with 397 participants between the ages of 15 and 26; 12% of them stated that they were hospitalized due to menstrual pain, and 69.6% of participants considered that dysmenorrhea affected their mood [40]. Dysmenorrhea affects the academic performance of female students through loss of attention and inactivity in classes, absenteeism, the inability to do homework, and poor results in exams [41]. Sahin et al. reported that anxiety and depression levels were higher in adolescent girls with dysmenorrhea compared to those without this condition and were more pronounced if the pain was more intense [42]. Mizuta et al. evaluated the impact of dysmenorrhea on patients’ quality of life using the 26-item World Health Organization Quality of Life scale (QoL) and observed that the QoL score was statistically significantly lower in the case of severe menstrual pain [43]. Dysmenorrhea is also an economic issue because patients with this condition frequently need health care services and take large amounts of medication to reduce pain every month [44].

Table 2 shows the results of several studies that evaluated the impact of dysmenorrhea on patients’ quality of life.

## 3. Endometriosis and Patients’ Quality of Life

Endometriosis is defined as the presence of endometrial tissue (glands and stroma) outside the uterine cavity [51]. Endometriosis lesions can be superficial (peritoneal or ovarian) or deep infiltrating. The etiology of endometriosis is not completely known. There are several theories that attempt to explain the presence of endometrial tissue outside the uterus, such as retrograde menstrual flow or Müllerian remnants that have not migrated and differentiated in fetal life [52,53].

In the United States and Canada, the prevalence of endometriosis varies between 5 and 15% among women of reproductive age. Although many patients are asymptomatic, endometriosis can cause unpleasant symptoms such as chronic pelvic pain in 6 to 10% of cases, infertility, dyspareunia, dysmenorrhea, or dyschezia [54]. Approximately one third of patients with endometriosis have infertility [55]. Approximately 2–4% of menopausal women have endometriosis. The decrease in estrogens after menopause can improve symptoms in these patients, but they can present non-specific symptoms that require additional and repeated investigations, because in this period of life, the presence of malignancies is suspected [56].

The non-specific symptomatology makes diagnosis difficult. It was reported in a study that included 1418 women of reproductive age, aged 18–45 years, that diagnosis was made with a delay of 6.7 years from the onset of symptoms to the surgical and pathological confirmation of endometriosis lesions. All this time, the women’s quality of life was affected by the presence of symptoms. Daily activities were affected, and working hours and productivity were reduced, which led to high costs for each woman [57]. Endometriosis is also an economic problem because in the United States annual costs of USD 16,573 have been reported for each woman diagnosed with this condition, three times more than in the case of women without endometriosis [58].

Endometriosis can have an impact on health-related quality of life (HRQoL), with negative effects on social, psychological, or physical aspects of life for patients. In a meta-analysis by Sima et al., it was observed that the 36-item survey generic questionnaire (SF-36) represents an efficient tool for evaluating the quality of life of patients with endometriosis. They reported that physical functioning was the most affected parameter but showed significant improvements after surgical or hormonal treatment [59]. Specific questionnaires were developed to measure the impact of endometriosis on quality of life. The Endometriosis Health Profile (EHP-30) includes 30 questions divided into five categories: pain, social support, control and powerlessness, self-image, and emotional well-being [60]. The EHP-30 was implemented in 2001 and is currently available in over 56 certified translations. A systematic review that included 139 studies using the EHP supported the validity, reliability and wide acceptability of this questionnaire [17]. The Endocare questionnaire (ECQ) measures the patient-centeredness of endometriosis, meaning that women can evaluate their experiences with health services and certain aspects can be improved in specific clinics [61,62]. In a systematic review by D’Alterio et al., which included 37 studies, it was observed that SF-36 and EHP-30 were the most frequently used questionnaires for evaluating the impact of endometriosis on quality of life. In addition, both medical and surgical treatment have been reported to reduce pain and improve quality of life, so treatment methods must be personalized and take into account the patient’s wishes [63].

In Table 3, the results of studies evaluating the impact of endometriosis on patients’ quality of life are presented.

## 4. Infertility and Patients’ Quality of Life

Infertility is a common problem nowadays, and it is associated with the presence of diseases of the reproductive system, but it is also closely related to social and psychological factors. Infertility is defined as the impossibility of a couple to conceive after 12 months of regular sexual intercourse. Two types of infertility have been described: primary (a couple without pregnancy history) and secondary [69]. The prevalence of infertility worldwide varies between 12.6% and 17.5%, which means approximately one in six couples. Higher rates have been reported in America, Europe, Western Pacific, and Africa [70].

Negative emotional experiences are common among infertile couples, and most of the time, quality of life is low. These things can also be influenced by the culture of a country, by traditional social concepts, by access to counseling and health services, and by the high cost of treatments. Resilience is very important in the impact of infertility on the quality of life of patients [71]. Infertility is also an economic problem. Rural/urban residency areas, monthly income, and educational status influence the impact of infertility on quality of life. Women with infertility who live in rural areas present a lower quality of life from an economic, psychological, and emotional point of view [72].

Infertility can influence the quality of life of patients through the appearance of difficulties in marital life, dysfunction in sexual relationships, or psychological problems. A meta-analysis by Nik Hazlina et al. evaluated the psychological impact that infertility can have on women. Compared to the general population, women with infertility have a 60% higher risk of psychological distress, a 60% higher risk of anxiety, and a 40% higher risk of depression [73]. In a study that included 100 nulliparous patients with infertility between 20 and 38 years old and 100 patients in the control group, the impact of infertility on patients was evaluated using the WHOQOL-BREF questionnaire and the Depression Anxiety and Stress Scale (DASS-21). A significantly lower quality of life in infertile women was reported in the physical, social, psychological, and environmental domains (*p* < 0.001). The number of women with anxiety, stress, and depression was significantly higher in the group of infertile women [74].

The FertiQoL (Fertility Quality of Life) questionnaire represents a specific, valid, and reliable tool used to evaluate quality of life among both women and men experiencing infertility. The impact of infertility on the quality of life of patients can be affected by the duration and type of infertility, as well as by the reason for infertility [75]. An increase in the duration of infertility was associated with a decrease in FertiQoL scores. Patients with infertility can feel inferior in the relationships they have with people who have children or may experience hopeless feelings [76]. A study by Dourou et al., which included 101 couples with fertility problems and used The Demographic Information and Medical History Questionnaire and the FertiQoL questionnaire, reported that the higher the level of stress and anxiety, the greater the decrease in quality of life observed among patients with infertility. The number of conflicts in infertile couples has increased. Infertility can cause problems with social interactions. Some women with infertility present behavioral or cognitive problems [77]. A study by Wdowiak et al. included 1200 patients treated for infertility who completed the FertiQoL questionnaire. Women who were treated without the use of assisted reproductive technology (non-ART) had significantly lower scores on four subscales of FertiQoL (emotional, partnership, biological, and attitude towards treatment) compared to patients who received IVF. In addition, quality of life was influenced by whether the women had children from other relationships, as well as professional activity [78]. Before and during treatment, higher degrees of general distress and anxiety were experienced by women when the infertility was of only a female cause. These data should be considered by health service providers, as they show the importance of psychological support in cases of infertile couples [79].

## 5. Menopause and Patients’ Quality of Life

Menopause is the permanent cessation of menstruation for a continuous period of at least 12 months. The reduction in the serum level of estrogen determines the appearance of menopause symptoms: anxiety, depression, sleep problems, changes in metabolism, urogenital symptoms, sexual dysfunction, musculoskeletal symptoms, and vasomotor symptoms [80].

In a study by Baral et al., approximately 51.4% of menopausal women presented with symptoms that decreased quality of life. Educational attainment, treatment for the health problems, alcohol intake status, and physical activity were significant factors in determining the quality of life of these women [81]. In addition to the decrease in quality of life, women with menopausal symptoms presented higher health care utilization and work impairment compared to women who did not have menopausal symptoms. Health outcomes were associated with anxiety and depression [82]. Practicing regular physical exercises can lead to an increase in quality of life in menopausal women, while being overweight has the opposite effect [83,84]. The Menopause Rating Scale (MRS) is used to evaluate the severity of menopause symptoms in three categories: urogenital, vasomotor, and psychological symptoms. A negative correlation was observed between MRS total scores and the scores for QoL evaluated using WHOQOL-BREF. The level of monthly family income and educational level of menopausal women were positively correlated with QoL total scores [85]. Menopausal women are a well-represented group from an economic point of view in the workplace. A systematic review showed that age, type of work, educational level, working environment, menopausal symptoms, permanent place of residency, comorbidities, and mental factors affect quality of life at work [86].

The MENQOL questionnaire (Menopause-specific Quality of Life Questionnaire) is a tool used to evaluate how menopause influences quality of life. It involves the evaluation of 29 symptoms experienced, divided into four domains: vasomotor, psychological, physical, and sexual [87,88]. In a study by Barati et al. that included 270 postmenopausal women, MENQOL was used to assess the prevalence of menopausal symptoms and the factors involved in affecting the quality of life of postmenopausal women. It was observed that vasomotor symptoms were the most frequent: hot flushes or flashes (75.9%) and night sweats and sweating (59.3%). It has been reported that quitting smoking and increasing one’s level of physical activity or intake of omega 3 fatty acids lead to an improvement in quality of life in menopausal patients [89]. The results of a systematic review by Jenabi et al. showed that SF-36 and MENQOL are the most used questionnaires for evaluating the quality of life of menopausal patients [90].

## 6. Pelvic Organ Prolapse and Patients’ Quality of Life

Pelvic organ prolapse (POP) has an incidence ranging from 2 to 50%. This is defined as a change in the pelvic organs to descend from the anatomical position towards the vaginal introitus or beyond it, due to the loss of support provided by muscles and connective tissue. Elderly women may experience various symptoms such as pelvic pressure, urinary and bowel dysfunction, vaginal bulge, and sexual dysfunction. POP represents a major health problem, especially in underdeveloped countries. Primary and secondary POP prevention measures should be part of routine practice for health care providers [91,92]. The prevalence of surgeries for prolapse varies a lot and reaches a peak between 60 and 69 years [93]. Factors associated with the appearance of POP are older age, familiar history of POP, obesity, high parity, forceps delivery, home delivery, prolonged labor, chronic constipation, performing heavy work, vaginal trauma, and a history of perineal tear. Women with POP report impaired daily activity and low productivity. POP affects sexual, physical, psychological, and social functions in women [94].

POP affects the daily activities of women; despite this, it often remains untreated. In a study that included 521 patients clinically diagnosed with POP, 60.8% of them stated that the symptoms associated with POP moderately or severely affected their quality of life and 47.8% of them considered that daily activities were moderately or severely affected in terms of their homework or job, hygiene, and social life. A significant percentage of women with POP (44.3%) claimed that they had had symptoms for more than 5 years, and 17.5% stated that they also had symptoms associated with urinary incontinence [95].

Even if POP does not represent a life-threatening condition, it is associated with significant morbidity. Women with POP show increased distress and a low quality of life. The intensity of the symptoms and the evaluation of quality of life among women with POP are very important for the establishment of treatment and for the development of new treatment methods. The Pelvic Floor Impact Questionnaire short form (PFIQ-7) and the Pelvic Floor Distress Inventory short form (PFDI-20) represent specific questionnaires that evaluate the symptoms of pelvic floor disorders and their impact on quality of life [96]. The Prolapse Quality of Life Questionnaire (P-QoL) represents a valid, feasible, and simple method for evaluating POP symptoms and the impact they have on patients’ quality of life. The questionnaire includes 20 questions grouped into nine domains that cover general health, physical and social limitations, personal relationships, prolapse impact, sleep/energy disturbance, emotional problems, and roles limitations, as well as the evaluation of symptom severity [97]. Women diagnosed with POP may have limitations regarding their physical activities, household, job, ability to travel, social life, relationships with partners, and sexual activity. These women may show symptoms of depression, anxiety, and nervousness, feel bad about themselves, and have impaired sleep [98,99].

The prevalence of female sexual dysfunction has been estimated at 30–50% in the general population, and among women with pelvic floor disorders, it has been reported to be higher, approximately 50–83%. The reduction in sexual activity in these women may be due to the opinion they have about the appearance of the genital area in pelvic organ prolapse, coital incontinence, and dyspareunia in urinary incontinence or the fear of dirt in anal incontinence. Pelvic floor muscle training and native tissue repair in POP are associated with an improvement in sexual function. Vaginal mesh repair and levatorplasty could increase the incidence of dyspareunia, while subtotal hysterectomy do not show an improvement in sexual function compared to total hysterectomy. Contradictory effects on sexual function have been reported with the use of vaginal pessaries in POP [100]. Pessaries are a conservative treatment method for POP and can be used as a first line of treatment, especially in patients with associated pathologies (diabetes mellitus or cardiovascular disease). The ring pessary is easy to remove and reinsert, has a good rate of prolapse reduction, and is the most common type of vaginal pessary. Vaginal erosion and discharge are adverse effects that may cause patients to stop using pessaries. Some patients choose to have surgical treatment for POP after a period of using pessaries [101]. There are two main categories of pessaries: long-term wear pessaries, which are removed only during specialist visits, and daily control pessaries, which are removed and reinserted daily. The choice of pessary type depends on socioeconomic, demographic, and functional health-related factors. It has been observed that sexually active patients have chosen to use the daily control cube pessary [102]. Vaginal pessaries have been associated with improved quality of life, sexual function, vaginal symptoms, and mental health [103,104]. On the other hand, it has been reported that the use of vaginal pessaries is not associated with changes in the sexual function of patients. Ring pessaries are compatible with sexual activity [105]. If partners find the pessary uncomfortable or impairing sexual function, it can be removed and reinserted as needed [106]. In a review by Wharton et al., the results showed that there was no deterioration or negative change in sexual function in women with POP who used pessaries as a treatment method [107]. In a study by Carlin et al. that included 130 postmenopausal women, a significant improvement in the “sexual function” domain was observed after pelvic floor surgery compared to the use of vaginal pessaries for three months [108].

Pelvic floor therapy is a conservative alternative treatment for POP, and it involves performing exercises to improve control, strength, and relaxation of the pelvic floor muscles. This therapy may include home exercise programs, biofeedback or electrical stimulation, behavioral education, or manual therapy [109]. Pelvic floor physiotherapy has been associated with increasing pubococcygeus tonicity with a reduction in symptoms associated with POP, increasing quality of life, and improving sexual function in these patients. It has been observed that the combined use of physiotherapy techniques (Kegel exercises and electrostimulation) can cause significant changes in sexual function such as increased libido or arousal and improvements to orgasm and dyspareunia [110]. Pelvic floor muscle training can improve sexual function by increasing the strength and endurance of the pelvic floor muscles [111]. Electrical stimulation biofeedback therapy uses electrical impulses to stimulate pelvic nerve fibers in order to improve the function of the pelvic neuromuscular system. Pelvic floor exercises combined with electrical stimulation biofeedback therapy can have positive effects on POP symptoms, improve quality of life, and improve sexual function [112].

The Pelvic Organ Prolapse/Incontinence Sexual Questionnaire—International Urogynecologic Association (IUGA) Revised (PISQ-IR) evaluates the impact of symptoms associated with pelvic floor disorders on the sexual function of women who are or are not sexually active and includes arousal/orgasm, condition-specific, desire-related, partner-related, condition impact, and global quality rating questions [113,114]. A study by Rusavy et al. included 333 patients with POP stage ≥2 who underwent laparoscopic sacrocolpopexy and completed the PISQ-IR before and one year after surgery. It was found that the prevalence of dyspareunia decreased and the general scores for sexual functions showed improvement one year after surgery [115].

## 7. Urinary Disorders and Patients’ Quality of Life

Urinary incontinence (UI) is defined as the involuntary leakage of urine. It affects millions of people worldwide and it has underestimated negative effects on personal well-being. It is more common in women than in men. Its prevalence cannot be assessed correctly; it is estimated that approximately one in four people may present with urinary incontinence throughout their lives. Most of the time, patients consider that the involuntary leakage of urine is something normal as they become older, and they are ashamed to recognize its presence [116]. Urinary incontinence can have negative effects on patients’ quality of life through its impact on sexual function, social life, and relationships with friends. Patients with urinary incontinence may have unpleasant feelings (“wet”, “dirty”, “smelly”) and may thus limit their physical or social activities such as shopping, dancing, or going out with friends. Patients can also present with low self-esteem, decreased sexual desire, a desire for isolation, and signs of depression [117].

Three types of UI have been described: urgency urinary incontinence (UUI), stress urinary incontinence (SUI), and mixed urinary incontinence (MUI). SUI involves the involuntary loss of urine when abdominal pressure increases during exercise, laughing, or coughing. UUI involves strong micturition urgency unrelated to the level of bladder fullness that causes urine loss. MUI combines symptoms of the other two types of urinary incontinence [118]. The quality of life of women with UI decreases with the severity of the symptoms and varies according to the type of UI. It has been observed that UUI decreases the quality of life of women the most. There are also other factors that influence the quality of life in women with UI, such as age, associated medical conditions, treatments, and duration of symptoms. However, a small proportion of women seek health care for this pathology [119]. In a study by AlQuaiz et al., women with SUI or UUI were more likely to report moderate/severe mental distress. Meanwhile, women with UUI or severe urinary distress reported more frequent or decreased self-esteem [120]. The results of a study by Chow et al., which included 4208 women with SUI over 40 years of age, showed that frequent episodes of SUI were associated with a more severe quality of life impairment in mental and physical health domains [121]. Urogynecological procedures for the treatment of UI in women do not always improve quality of life. Age and time since surgery are important for determining quality of life. Most of the time, surgery in elderly patients does not bring about beneficial effects [122].

## 8. Polycystic Ovarian Syndrome and Patients’ Quality of Life

Polycystic ovary syndrome (PCOS) is the most common endocrine disorder in women of reproductive age. This is characterized by the presence of hyperandrogenism and oligoovulation or anovulation, which can cause infertility and metabolic disorders [123,124]. PCOS symptoms produce high stress and can have negative psychological or sexual effects and can affect social well-being [125]. Mental distress in these women occurs due to the presence of irregular periods, physical appearance, or infertility and thus can affect quality of life. Increased BMI, educational status, menstrual irregularities, and marital status represent factors that influence quality of life in patients with PCOS [126]. Women who have severe or more severe symptoms of PCOS have a lower quality of life compared to those who have mild symptoms. Women who consider that they have a lower quality of life face a feeling of a lack of control over the disease, do not accept their physical appearance, and present depressive symptoms [127].

The PCOS Health-Related Quality of Life Questionnaire (PCOSQ) is a specific tool used to evaluate the negative impact of PCOS on the quality of life of patients. The PCOSQ has five domains: emotions, menstrual problems, body hair, weight, and infertility problems [128,129,130]. In a systematic review by Bazarganipour et al., it was shown that menstruation and hirsutism were the domains that most affect quality of life in PCOS patients [131]. Women with irregular periods or hirsutism may feel less feminine than other women. Up to 90% of women diagnosed with PCOS consider facial hair to be a problem (reporting feelings like “different”, “unfeminine”, “strange”), and their physical appearance can make young females feel less sexually attractive. Anxiety, depression, fatigue, and sleep problems can affect the quality of life of women with PCOS [132].

Women with PCOS had both a low quality of life and a low satisfaction with life, which were influenced by socioeconomic standing, the time since PCOS diagnosis, and BMI. In addition, quality of life was influenced by age and professional activity, and satisfaction with life was influenced by having children [133]. It was observed that the practice of regular physical exercises had beneficial effects on the quality of life of PCOS patients by improving the ovulation rate, leading to reduction in mental disorders, increasing menstrual regularity, or leading to weight loss [134]. Another essential element for improving the quality of life of PCOS patients is an adequate diet. A low-calorie diet is recommended, with a low intake of high-glycemic-index carbohydrates and saturated fatty acids, but with an increase in the intake of polyunsaturated fatty acids and fiber [127]. The most effective diet in reducing PCOS-related symptoms has been shown to be the Mediterranean diet, which includes large amounts of omega-3 fatty acids, which maintains low blood glucose and triglyceride levels but at the same time reduces androgen bioavailability. Other diets that have the potential to improve symptoms associated with PCOS are the high-fat ketogenic diet and lactose-free diet [135]. In addition to dietary changes and regular physical activity, other factors have been identified that are useful in improving the quality of life of patients with PCOS, such as behavioral interventions, reducing alcohol intake, and smoking, but also psychological approaches (management of anxiety, depression, sexual dysfunction, or negative self-image), optimizing sleep, and traditional, complementary, and integrative medicine (inositol supplementation, vitamins, and minerals) [136].

## 9. Uterine Fibromatosis and Patients’ Quality of Life

Uterine fibromatosis is the most common gynecological pathology in women of reproductive age, and it can be diagnosed in up to 70–80% of women during their lifetime. Abnormal uterine bleeding, pelvic pain, constipation, and infertility are the most common symptoms of uterine fibromatosis. It represents a real health and economic problem due to the high costs required for health care [137,138]. Uterine fibromatosis significantly affects the quality of life of patients, as well as mental health. It has been observed that all therapeutic interventions (surgical, medical, and radiological) used to treat uterine fibromatosis led to a decrease in the severity of the symptoms and improved quality of life and mental health [139]. In a study by Hervé et al., approximately 64% of participants stated that uterine fibromatosis moderately to severely affected their quality of life [140]. The results of a study by Koga et al. showed that anemia is a predictor factor for the decrease in quality of life due to the presence of uterine fibromatosis [141].

The Uterine Fibroid Symptom and Quality of Life Questionnaire (UFS-QOL) is a specific instrument with 37 questions that evaluates the symptoms associated with uterine fibromatosis, as well as their impact on quality of life. This questionnaire includes seven subscales: symptom severity, energy/mood, concern, activities, self-consciousness, sexual function, and control [142]. In a systematic review that analyzed the use of the UFS-QOL, it was observed that medical, surgical, or radiological treatment was associated with a decrease in severity scores for symptoms and with an improvement in quality of life [139].

Uterine fibromatosis can have a negative impact on the quality of life of women by decreasing productivity, affecting relationships and social life, and altering self-image and sexuality, as well as physical wellness [143]. It has been reported that uterine fibromatosis has a significant psychological impact on women, who may experience feelings of fear, anger, anxiety, or depression. In a study by Ghant et al., it was observed that more than half of the participants considered that they had no control over their disease and felt helpless. Women with uterine fibromatosis had lower self-esteem and considered themselves less attractive [144]. Also, uterine fibromatosis can affect professional activities. In a study by Borah et al., which included 968 participants with uterine fibromatosis, 28% of them stated that they were missing work due to uterine fibromatosis symptoms, and 24% of participants considered that the symptoms prevented them from reaching their career potential [145]. Another study with 841 participants with uterine fibromatosis reported that women had many concerns about uterine fibromatosis, such as the growth of fibroids (77%), the occurrence of possible complications (61), the need to perform a hysterectomy in the future (54%), or their evolution into cancer (53%). Approximately 52% of the participants were worried that uterine fibromatosis would affect their sex life or their relationship with their partner (42%) [146].

## 10. Abnormal Uterine Bleeding and Patients’ Quality of Life

Abnormal uterine bleeding (AUB) may affect up to 30% of women of reproductive age and is described as a change in the duration and frequency of menstruation or the amount of blood lost (heavy menstrual bleeding (HMB)). It may occur as a result of a functional or anatomical abnormality or a systemic disease. Abnormal uterine bleeding has been observed to have a negative effect on the quality of life of patients and can cause insufficiency in the workplace, and its treatment can be a problem for health services. Menorrhagia has been associated with negative effects on physical, psychological, and social health, family life, work, and daily activities [147]. The severity of menorrhagia is associated with the extent of negative effects on patients’ quality of life [148]. HMB has also been associated with affecting energy, mood, self-consciousness, and sexual function [149]. The results of a study using the SF-36 showed that women with HMB had significantly lower scores in all domains for quality of life compared to women with normal menstrual bleeding [150]. HMB can cause iron deficiency anemia, and this has been significantly associated with shortness of breath or feeling tired during the menstrual period [151].

Adenomyosis is one of the most common causes of HMB and is characterized by the presence of endometrial tissue in the myometrium. Its prevalence increases with age and is higher in perimenopause or multiparous women. However, it can also be diagnosed in adolescents or young women who present with HMB or dysmenorrhea. HMB is underestimated and underreported in adolescents but can have a negative effect on their quality of life [152]. In one third of cases, adenomyosis is asymptomatic, but, depending on the intensity of symptoms (AUB, dysmenorrhea, dyspareunia, possibly infertility), it can affect the quality of life of patients. This condition can be associated with an early onset in adolescence, and the intensity and severity of symptoms can increase over time. Adenomyosis is often associated with endometriosis, a common cause of chronic pelvic pain. Early non-invasive diagnosis (ultrasound or magnetic resonance imaging) should be encouraged for optimal management of this pathology to improve long-term outcomes and patients’ quality of life [153]. Adenomyosis can cause disabling symptoms and is associated with increased health care utilization. In young women, medical treatment is the first line of treatment. But in the long term, these therapies are limited compared to hysterectomy [154]. The quality of life of patients with adenomyosis is low and is associated with negative effects on mental and physical health. Patients with adenomyosis are at higher risk of depression and anxiety compared to patients with uterine fibroids [155]. Adenomyosis has been associated with decreased productivity at work and impairment in both daily and professional activities [156].

## 11. Other Gynecological Pathologies and Patients’ Quality of Life

From menarche to menopause, women face a series of gynecological pathologies that can influence their lives in many aspects and can have repercussions in several areas: school, work, sexuality, and psychology. In Table 4, we briefly present the negative effects of the gynecological pathologies presented above.

In addition to the gynecological pathologies presented above, there are other conditions or symptoms related to the genitourinary system that influence various aspects of patients’ quality of life.

Vulvovaginitis can be considered a public health problem, considering the discomfort it creates and the alterations to well-being and patients’ quality of life [157]. Bacterial vaginosis is one of the most common conditions in women of reproductive age. Its presence has been associated with a negative impact on sexual, physical, and mental health [158]. Recurrent vulvovaginal candidiasis (RVVC) affects millions of women throughout their lives. This pathology requires special attention because it has been associated with a decrease in patients’ satisfaction with their health and their perception of their quality of life. RVVC had negative effects on physical and psychological well-being but also on sexual activity [159,160].

Urinary tract infections (UTIs) are very common among sexually active women of reproductive age, and effects can range from physical symptoms to social, financial, and psychological effects [161]. Patients with recurrent UTIs may experience anxiety generated by the sudden, distressing, and unpredictable nature of the symptoms generated by a new episode of UTI. Social life may be impaired, and signs of depression may appear. An even more significant negative impact is observed among women with UI, whose symptoms are exacerbated by urinary UTIs. It has been observed that the use of methods of prevention for the occurrence of new episodes of UTIs can improve quality of life among these patients [162]. It was observed that urinary tract infection symptoms were associated with a series of negative emotions (helplessness and fear) and influenced relationships with others, daily activities, and sleep. Treatment failure was associated with anger and frustration [163].

Chronic pelvic pain is a multifactorial and complex condition. It affects approximately 14% of women throughout their lives and involves high costs to the health system, as well as impairments in functional capacity and quality of life. Chronic pelvic pain has been associated with gynecological, urological, gastrointestinal, musculoskeletal, neurological, and psychological pathologies. The gynecological conditions most associated with chronic pelvic pain are endometriosis, adenomyosis, and fibroids [164], which have been discussed extensively in this review. The use of the WHOQOL-BREF questionnaire showed that the presence of chronic pelvic pain was associated with lower scores in the psychological, social relationship, physical health, and environment domains [165].

## 12. Conclusions

Nowadays, quality of life is an important topic, and we must pay more attention to the factors or conditions that can negatively affect it. From adolescence to postmenopause, women face a series of gynecological conditions that can significantly reduce their quality of life through negative psychological and social effects, by affecting relationships, sexual life, and academic or professional activities and by limiting daily activities. From the effects of dysmenorrhea that occur from the teenage years until the onset of menopause symptoms, women frequently present with a series of gynecological disorders such as endometriosis, uterine fibromatosis, polycystic ovary syndrome, pelvic organ prolapse, or urinary incontinence. We conducted a literature review to provide a general, descriptive, and comprehensive perspective on this topic. The flexible approach of a narrative review may result in relevant studies not being included, and variations due to population-based factors (ethnicity, culture, or religion) may not be fully discussed.

Understanding the negative effects that these conditions have on quality of life can contribute to more efficient and personalized management of cases. Health care providers should be aware of the impact of gynecological pathology on quality of life. They should have a focused discussion on this topic with patients, so that the most appropriate treatment method can be chosen. The quality of life of patients should be a priority for health care providers.

## Figures and Tables

**Table 1:** Questionnaire translation or validation.

Questionnaire	Abbreviation	Translation or Validation
World Health Organization Quality of Life scale	WHOQOL-BREF	Validated in over 40 languages (e.g., United State of America, Japan, Netherlands, Australia, Poland, India, Bangladesh, Thailand, Croatia) [3,12]
Medical Outcomes Study Short Form-36 Health Survey	SF-36	Evaluated in more than 50 languages [13]
Duke Health Profile	DUKE	Translated into more than 17 languages [14] and validated in France [15] and Vietnam [16]
Endometriosis Health Profile	EHP-30	A total of 56 certified language versions [17]; validated in Portuguese [18], Turkish [19], and Swedish [20]
Fertility quality of life questionnaire	FertiQoL	Translated into more than 20 languages [21,22,23]
Menopause-specific Quality of Life Questionnaire	MENQOL	Validated in Greek [24], Chinese [25], and Persian [26] languages
Prolapse Quality of Life Questionnaire	P-QoL	Validated in English, Japanese, Italian, German, Turkish, traditional Chinese, and Persian languages [27]

**Table 2:** Dysmenorrhea and patients’ quality of life.

Authors (Year)	Number of Participants	Prevalence of Dysmenorrhea	Mean Age of Participants (Years)	The Effects of Dysmenorrhea on the Quality of Life
Hailemeskel et al. [45] (2016)	440	85.4%	20.57 ± 1.36	80.0%—school absence
				21.0%—inability to do homework
				66.8%—loss of concentration in class
				47.4%—reduced participation in class
				37.8%—limited sport participation
				31.7%—limitations in going out with friends
Esan et al. [40] (2024)	397	69.8%	19.73 ± 1.1	50.2%—affected routine work
				38.8%—disturbed sleep
				44.8%—social withdrawal
				53.8%—restricted physical activities
				45.8%—have been late to class
Fathi et al. [46] (2022)	298	83.6%	20.32 ± 3.19	25.5%—affected daily activities
				10.4%—affected personal care
				8.4%—problems with mobility
Unsal et al. [47] (2010)	623	72.7%	20.8 ± 1.8	Significantly lower scores in Short Form-36 (SF-36) domains in students with dysmenorrhea: - Physical functioning; - General health perception; - Bodily pain; - Role—physical; - Vitality.
Mohamed et al. [48] (2013)	1092	78.8%	16.8 ± 0.87	45.2%—having problems with family
				63.4%—living apart from their family
				67.9%—unwilling to talk with friends
				71.1%—not comfortable in relationships with friends
Hashim et al. [49] (2020)	376	80.1%	20.97 ± 1.49	SF-36 domains statistically significantly affected by dysmenorrhea: - Physical health; - Health changes; - Emotional health; - Pain.
Chia et al. [50] (2013)	240	80.0%	20.1 ± 1.4	26%—sleep disturbance
				3%—hospital admission
				36%—negative impact on psychosocial well-being
				75%—study disturbances and/or reduced ability to concentrate

**Table 3:** Endometriosis and patients’ quality of life.

Authors (Year)	Number of Participants with Endometriosis	Questionnaire	The Effects of Endometriosis on Quality of Life
Warzecha et al. [64] (2020)	246	EPHect Patient Questionnaire (EPQ)	70.2%—reduced physical activity
			69.5%—reduced sexual activity
			39.5%—reduced professional activity
			15.1%—diagnosed with depression
Gete et al. [65] (2023)	615	SF-36	Worse scores on the SF-36 than patients without endometriosis: - Physical functioning; - Role physical; - Bodily pain; - General health; - Vitality; - Social functioning; - Role emotional; - Mental health; - Physical Health Component Scale; - Mental health components.
Bień et al. [66] (2020)	309	-WHOQOL-BREF -Acceptance of Illness Scale (AIS)	- Overall QoL score: 3.30 - Overall perceived health score: 2.37 - The highest QoL scores: psychological domain - The lowest QoL scores: physical domain - Moderate level of illness acceptance (24.64)
Missmer et al. [67] (2022)	743	Original survey	Patients ‘somewhat agreed’ or ‘strongly agreed’ with the following: - Problems in their intimate relationships (83%); - A barrier to starting a family (58%); - Traveling less (59%); - Less outgoing (81%); - Limited the ability to maintain a healthy diet (55%); - Endometriosis-associated symptoms led to the use drugs, cigarettes, or alcohol (43%); - Less positive about the future (80%); - Feeling of not having reached their full potential (75%).
Tiringer et al. [68] (2022)	115	EHP-30	Preoperative and 6–10 weeks postoperative values were compared: - Significant improvements in the following domains: “pain”, “self-determination”, “emotional health”, “social environment”, and “self-image” (*p* < 0.001), especially women with deep-infiltrating endometriosis with or without ovarian endometrioma.

**Table 4:** The gynecological pathologies and patients’ quality of life.

Gynecological Pathology	School	Work	Sexuality	Psychology
Dysmenorrhea	Frequent absences during menstrual cycles Negative impact on school performance Loss of concentration in class Inability to complete homework	Frequent absences during menstrual cycles	Reduced sexual desire due to pain	Sleep disturbance
				Negative impact on psychosocial well-being
				Anxiety
				Depression
Endometriosis	Rare, but possible in severe case	Reduced professional activity Reduced working hours and productivity	Reduced sexual activity	Depression
				Anxiety
				Affected mental health
				Sleep disturbance
Infertility	Not applicable	Rare	Difficulties in marital life Dysfunction in sexual relationships	Psychological distress
				Anxiety
				Depression
Menopause	Not applicable	Variable	Vaginal dryness Decreased libido	Anxiety
				Depression
				Sleep problems
Pelvic organ prolapse	Not applicable	Limitations of professional activity	Limitations in sexual activity Discomfort during sexual intercourse	Low self-esteem
Urinary disorders	Not applicable	Variable depending on the intensity of symptoms	Negative impact on sexual function Decreased sexual desire	Low self-esteem
				Desire for isolation
				Depression
Polycystic ovarian syndrome	Indirect (due to depression or fatigue)	Rare	Negative impact on sexual function Feelings like “different”, “unfeminine”	Negative psychological effects
				High stress
				Feeling of lack of control over the disease
				Anxiety
				Depression
				Fatigue
				Sleep problems
Uterine fibromatosis	Rare	Decreasing productivity (due to pain or heavy bleeding)	Negative impact on sexual function Dyspareunia	Altered self-image and physical wellness
				Fear
				Anxiety
				Depression
